# Ethical and Statistical Considerations in Models of Moral Judgments

**DOI:** 10.3389/frobt.2019.00039

**Published:** 2019-08-16

**Authors:** Torty Sivill

**Affiliations:** Computer Science Department, University of Bristol, Bristol, United Kingdom

**Keywords:** morality, assumptions, utility theory, decision-making, artificial, ethics, deontology, utilitarianism

## Abstract

This work extends recent advancements in computational models of moral decision making by using mathematical and philosophical theory to suggest adaptations to state of the art. It demonstrates the importance of model assumptions and considers alternatives to the normal distribution when modeling ethical principles. We show how the ethical theories, utilitarianism and deontology can be embedded into informative prior distributions. We continue to expand the state of the art to consider ethical dilemmas beyond the Trolley Problem and show the adaptations needed to address this complexity. The adaptations made in this work are not solely intended to improve recent models but aim to raise awareness of the importance of interpreting results relative to assumptions made, either implicitly or explicitly, in model construction.

## 1. Introduction

Modern artificial intelligence (AI) is being applied to contexts that have dramatic social implications. We are seeing the introduction of AI in potential life or death situations, the criminal judiciary system and positions of care for those most vulnerable in society. As computers become increasingly prevalent, the subject of designing intelligent systems that function responsibly is increasingly important (Moor, 2006). A recent report published by the House of Lords Select Committee on Artificial Intelligence on 16th April 2018 recognizes the importance of ethical consideration and recommends concentrating funding in this area (Artificial Intelligence, 2018).

The Select Committee highlights autonomous vehicles (AV) as an area requiring urgent consideration, specifically how AV make ethically sensitive decisions. The recent fatal accident in Arizona involving Uber's unmanned vehicle and a pedestrian fueled doubt over the ethicality these vehicles. In 2017, the UK Government pledged a significant proportion of its 70 million AI budget to having fully automated cars in use by 2021. However, in early 2018, the UK government announced a 3-year review to assess the risks of AV before they are tested on British roads.

The challenge of designing intelligent systems capable of making moral decisions is captured by the argument over three types of knowledge: explicit, implicit and tacit. Explicit knowledge is the type of knowledge that can be extracted from an individual over suitable enquiry (Dummett, 1991). Implicit knowledge, on the other hand, is made up of “what we know but aren't aware of” (Masters, 1992). Tacit knowledge refers to knowledge that is unable to be articulated and “tied to the senses” (Nonaka and von Krogh, 2009). There is much debate in academia surrounding the origins of moral principles, particularly concerning the extent to which explicit knowledge impacts moral judgment. The principle of phenomenalism, coined by Kohlberg et al. (1983), denotes the line of enquiry that considers a behavior as moral only if it is motivated by an explicit moral principle. However, critics argue that this argument isolates moral behavior that involves no prior deliberation, like the split second decision we may make when swerving a car. Narvaez and Lapsley (Narvaez and Lapsley, 2005), argue that much of moral behavior is automatic and choose to consider morality from a psychological, instead of a philosophical, perspective. They argue that much of moral behavior occurs unconsciously or tacitly, as opposed to explicitly. Much literature has been dedicated to the problem of extracting and understanding tacit knowledge from human behavior (Wagner and Sternberg, 1985; Nonaka and von Krogh, 2009). However, creating definitions over tacit knowledge is extremely difficult: how do we explain what we do not understand? This seemingly unsolvable paradox significantly affects the field of engineering moral machines, how are we to bridge the semantic gap between human morality and codifying ethical principles?

The field of ethical AI works to regulate artificial intelligence, ensuring applications are socially responsible. Machine morality extends ethical AI to consider the behavior of artificial moral agents, exploring how to engineer explicit moral reasoners (Allen et al., 2005). Wallach and Allen summarize approaches to engineering moral machines as either top-down or bottom-up (Allen et al., 2005). A top-down approach refers to the process of iteratively reducing a problem into individually solvable sub-tasks. Charisi et al. identify the most frequent form of top-down approach as system governance via a set of ethical rules (Charisi et al., 2017). This approach is adopted by Arkin et al.'s “ethical governor” (Arkin et al., 2012), a component of the ethical architecture for a military autonomous system. The ethical governor's purpose is to conduct an evaluation of the “ethical appropriateness” of an action prior to its completion by a robot (Arkin et al., 2012). The governor ensures a response is ethical by ensuring its non-membership of a pre-defined set of possible unethical outcomes where the “ethical appropriateness” of a response is determined by both utilitarian and deontological ethical theory. The ongoing academic debate over optimum ethical theories complicates top-down approaches and has been interpreted differently throughout the literature (Bendel, 2016; Dennis et al., 2016). Top-down approaches like these allow for a rigorous process of decision making (Charisi et al., 2017). However, the ambiguity of ethical theory makes it unclear as to whether top-down approaches dependent on specified ethical theory can be used in practice. Attention has, therefore, turned toward bottom-up approaches that do not require hard-coded ethical rules (Charisi et al., 2017).

Bottom-up approaches in engineering require a description of a problem and then the development of a method to find a solution in terms of its parameters (Charisi et al., 2017). In their general ethical analyzer, “GenEth,” Anderson and Anderson use machine learning to learn an ethical theory (Anderson and Anderson, 2014). GenEth was created alongside input from ethicists to help codify ethical principles in any given domain. In order to learn ethical principals GenEth uses inductive logic programming, a technique that learns relations based on First Order Horn Clauses. Contrastingly, Abel et al. use reinforcement learning to learn the most moral decision (Abel et al., 2016). Abel et al. use Markov Decision Processes as mechanisms to frame a decision problem with an associated reward function. Contrastingly, Dewey in his work, “Learning What To Value” (Dewey, 2011), argues that reinforcement learning can only learn preferences based on potential rewards. Dewey continues to present “expected observation utility maximization” as a mechanism that, unlike reinforcement learning, can be used to define agents with multiple final goals. Similarly, Boström in his recent work, “contemplating the feasibility of super-intelligence” (Boström, 2014), also questions the use of reinforcement learning in learning moral theory. Boström suggests that a sufficiently intelligent machine could maximize its reward by exploiting or “wireheading” its reward function (Boström, 2014). Although only applicable to some reinforcement learning scenarios, this vulnerability has shifted attention to Bayesian approaches.

Both Boström and Dewey suggest utility functions as a preferential way of ensuring AI learns about moral values instead of decision outcomes (Dewey, 2011; Boström, 2014). Bayesian learning is an alternative bottom-up technique that allows agents to make decisions that optimize a meta-utility function (Abel et al., 2016). In their paper, “Learning a Common Sense Moral Theory” (Kleiman-Weiner et al., 2017), Kleiman-Weiner et al. introduce a novel computational framework for learning moral theory. They first introduce a recursive utility calculus that captures welfare trade-offs in interactions between individuals and then use hierarchical Bayesian inference as a mechanism to understand the moral actions of individuals. Kleiman-Weiner et al. define abstract principles that capture simplified relationships between individuals and explain why a particular individual may act toward another. They propose a structured model where each individual's principles are generated from a prior, dependent on the group that the individual belongs to (Kleiman-Weiner et al., 2017). Building on this theory, the paper, “A Computational Model of Commonsense Moral Decision Making” (Kim et al., 2019), introduces a novel computational model of moral judgments in the AV domain. Kim et al.'s model describes moral dilemmas as utility functions that use abstract moral principles to compute trade-offs in decision making. Kim et al. use a Bayesian hierarchical model to categorize social structures of individuals and groups to show that individual moral preferences can be inferred as interpretable parameters from limited data.

Full human level moral agency is at present, technologically impossible. Machine morality today is therefore concerned with modeling a specific aspect of morality. This work is focused on the challenge of modeling human moral judgments. A greater understanding of how to successfully model these kind of decisions will form the basis for future work on moral machines, providing further insight into the philosophy behind human moral decisions.

We proceed by analyzing state of the art in this area that use models of human moral decision making to help define ethical behavior of autonomous systems (Kleiman-Weiner et al., 2017; Kim et al., 2019). We extend these works by first presenting potential pitfalls of their approaches, accompanying these hypotheses with experimental results. The focus of this project therefore, is not to propose a solution to machine morality but to highlight questions that must be addressed to drive future progress in this field.

We begin by examining the Moral Machine dataset (Awad et al., 2018), employed by Kim et al. in their model. The first of its kind to crowd-source morality on a large scale and question the ethicality of gamifiying this form of data collection. We present an alternative moral behavior dataset, collected by Faulhaber et al.'s autonomous vehicle study (Faulhaber et al., 2018), used in this work's model implementation. We continue to adapt the method of Kim et al. to build a model of moral decision making around the Faulhaber dataset, evaluating the model using Monte Carlo heuristics. Our model achieves 82% predictive accuracy. However, this paper's emphasis is not on quantified success but the issues our implementation has raised. We therefore challenge assumptions made by Kim et al. and highlight how these affect results.

Machine learning is an instance of inductive reasoning. As such, results generated by machine learning models can never be definitively proven right, they can only be proven wrong. Together with the “No-free-lunch” theorem (Wolpert, 1997), these results can only ever be interpreted relative to the assumptions made within the model. Historically, exciting results from machine learning experiments have been extrapolated beyond their studies. Reich et al. express how without sufficient evaluation of underlying assumptions there is no true meaning to results (Reich and Barai, 1999). This work shows the variability of models under different assumptions, intended to motivate rigorous evaluation in future work.

Prior assumptions have been identified as highly influential on statistical models. Kleiman-Weiner et al. highlight further exploration of optimal prior distributions for their model as an area for future work. We hypothesize that the prior distributions chosen by Kim et al. were chosen primarily for practicality and with further consideration, prior distributions could be found that more accurately reflect prior beliefs in moral theory. A difference between Bayesian reasoning and traditional methods is the inclusion of subjective beliefs about a probability in calculations. Bayesian inference is therefore composed of both current and prior knowledge which act together to make up the posterior (Van Dongen, 2006). The prior distribution plays a central role in Bayesian inference, particularly in circumstances where the likelihood does not dominate the posterior, i.e., the volume of data is limited. Prior specification becomes particularly challenging when using hierarchical models for Bayesian inference as these models require hyperparameters, each requiring a prior distribution (Gelman, 2006).

We conjecture that as a society we still know little about human morality, where it originates, and how it varies from individual to individual or group to group (Decety and Wheatley, 2015). We argue that the prior distributions chosen in the model of Kim et al. make assumptions about the moral preferences of the underlying data that do not reflect historic moral theory. The decision of Kim et al. to model individual weights and group norms as precisely normally distributed is particularly questionable. Firstly, considering group norms, do we expect these values to be closely concentrated around a central value? How do we expect these values to be correlated? Secondly, considering individual weights, do we consider these values to be closely concentrated around the group norm with low mass in distribution tails? Is this an accurate reflection of society? We present alternative prior distributions that attempt to address these issues.

Continuing our line of questioning, we consider utility calculus as a mechanism to capture human morality, and discuss the link between deontology and utilitarianism, showing through our implementation how deontological statements can be used as a prior for the utilitarian model to achieve greater predictive accuracy. We conclude by testing the model of Kim et al. on moral dilemmas that extend the Trolley Problem to consider more complex cases of morality.

## 2. Materials and Methods

### 2.1. MIT Moral Machine Dataset

The majority of historical work on moral machines is concerned with building theoretic models that formulate moral theory. However, if we are to succeed in building machines capable of making ethical decisions then these models need to be tested in real-world contexts. There is an urgent need for data that encapsulates information about how humans make moral decisions. Francis et al. identify moral dilemmas as key in helping researchers understand moral decision making and use their study to collect data about human decision making in the most famous of all moral dilemma thought experiments, the Trolley Problem (Francis et al., 2017). Francis et al. use virtual reality as an immersive technique to compare participants' responses to a variety of trolley problem scenarios. Contrastingly, researchers at MIT take a different approach to collecting these data. Their platform, The Moral Machine, is the first of its kind to “crowdsource morality” (Awad et al., 2018). As of October 2017 the platform had collected over 30 million responses from over three million respondents from over 180 countries from across the world (Kim et al., 2019).

The Moral Machine dataset has motivated further research in learning underlying moral preferences (Noothigattu et al., 2017; Kim et al., 2019). However, recent studies have warned of the dangers of using unqualified big data to hype experimental results (Lazer et al., 2014). As attention turns toward implementing real systems that make ethical decisions we need to ask the right questions concerning the data that these systems may be trained on. One particular concern with the data generated by the MIT Moral Machine platform is the gamification of data collection. Dergousoff and Mandryk raise questions surrounding the quality of results collected through a gamified approach to data collection when compared to traditional methods (Dergousoff and Mandryk, 2015). Similarly, Versteeg establishes gamification as a potentially “manipulative construct” (Versteeg, 2013), reinforcing our own opinions about the Moral Machine's data quality: are participants taking it seriously? What happens if someone gets bored in the middle of playing? Do people understand that this data is being used in academic research? We opt to select a new dataset which allows us to question the repeatability of Kim et al.'s results (Kim et al., 2019). Additionally, we select a dataset that has been collected in a traditional laboratory environment to avoid the ethical problems we have raised over the Moral Machine dataset.

### 2.2. Autonomous Vehicle Study Dataset

The data used for this project's implementation is from the German study “Human Decisions in Moral Dilemmas are Largely Described by Utilitarianism: Virtual Car Driving Study Provides Guidelines for Autonomous Driving Vehicles” (Faulhaber et al., 2018). This dataset is referred to as the “German Autonomous Vehicle” dataset for the remainder of the article. Faulhaber et al. conduct a set of experiments in which participants experience modified trolley problems as the driver of a car in a virtual reality environment. Participants are forced to make one of two decisions, choosing between the left or right lane when faced with obstacles. The obstacles consist of a variety of human-like avatars of different ages and numbers. Each participant is presented with one training track and five different experiment tracks. The car being driven by the participant is traveling at 36 miles per hour and the tracks range in length between 180 and 200 metres. The experiment tracks consist of five different environments: two mountain, a suburban and two city levels. In the city scenarios, the participant has to decide whether to swerve and mount the pavement or continue on the road. The presented avatars are: middle-aged man, old man, young boy, kneeling man, self (the participant). The avatars are all male, range in frequency and presented in random combinations. Prior to starting the experiment Faulhaber et al. ensure each participant is aware of the nature of the experiment and that they have signed a consent form, clarifying they are able to terminate the experiment at any time. Each participant is then presented with a sequence of training scenarios followed by 24 test scenarios across a combination of the five different test environments. Finally, the participants are asked a series of questions to ascertain age, gender and driving experience alongside a questionnaire containing high-level philosophical questions. 216 unpaid participants take part in the study and 201 participants complete the study.

To evaluate the success of Kim et al.'s model on the German Autonomous Vehicle Dataset we follow the method of Kim et al. (2019). However, for our implementation, the mapping between observable objects onto the abstract feature space is altered slightly due to the differences between the Moral Machine dataset and The German Autonomous Vehicle dataset. The characters represented by the German Autonomous Vehicle dataset are: driver of the vehicle (the respondent), man, old man, young boy, kneeling man and pedestrians. Our character vector is therefore represented as $\Theta_{y}\in {\mathbb{N}}^{K}$ where |*K*| = 6. Following Kim et al.'s approach, we believe that each of the six characters can be described by one or more of the following abstract principles: self-preservation, elderly, infancy, middle-aged, kneeling-down, pedestrian. Our implementation therefore uses the linear mapping *F*(Θ) = *A*′Θ where *A*′ is a 6x6 matrix.

### 2.3. Composing Utility Functions

Kim et al. choose to model the utility value of a resultant state as a linear combination of the features in the abstract dimension,

(1) $$\begin{array}{l} \mu \left(\Theta_{i}\right)=w^{⊤}F\left(\Theta_{i}\right), \end{array}$$

where Θ_0_ represents the state achieved from choosing not to switch lanes and Θ_1_ represents the state achieved by choosing to switch lanes. A respondent's decision to switch lanes, *Y* = 1, is represented by the sigmoid function of net utility of the two choices (Kim et al., 2019),

(2) $$\begin{array}{l} P\left(Y=1|\Theta \right)=\frac{1}{1+\exp^{-U\left(\Theta \right)}}, \end{array}$$

(3) $$\begin{array}{l} U\left(\Theta \right)=\mu \left(\Theta_{1}\right)-\mu \left(\Theta_{0}\right). \end{array}$$

### 2.4. Hierarchical Model

Kim et al. begin by considering *N* respondents from the dataset belonging to a group *g* ∈ *G* (Kim et al., 2019). Similarly to Kleiman-Weiner et al., Kim et al. state that this group could represent a country, culture or group within which customs and moral norms are shared. The moral principles of an individual *i* within group *g* are then drawn from a multivariate Gaussian distribution,

(4) $$\begin{array}{l} w_{i}\sim N_{D}\left(w^{g},\Sigma^{g}\right), \end{array}$$

where *w*^*g*^ denotes the mean values of the group *g* over *D* dimensions and the diagonal of the covariance matrix Σ^*g*^ represents the difference between members of group *g* over the abstract principles. The covariance matrix Σ^*g*^ also holds information about the strength of relationships individuals place between abstract principles. For example, if an individual values infancy highly they may also value the elderly highly. The covariance matrix allows the learner to quickly infer moral principles of one dimension after inferring those of a highly correlated dimension (Kim et al., 2019).

Let ***w*** = {*w*_1_, …, *w*_*i*_, …*w*_*n*_} be the set of moral principles of *N* respondents and the vector $\Theta =\left\{\Theta_{1}^{1},\ldots ,\Theta_{i}^{t},\ldots ,\Theta_{N}^{T}\right\}$ represent the resultant states of *i* respondents over *T* scenarios. The decision made by respondent *i* is represented by a random variable $Y_{i}^{t}$. From this foregrounding, Kim et al. are then able to define the posterior distribution,

(5) $$\begin{array}{l} P\left(w,w^{g},\Sigma^{g}|\Theta ,Y\right)\propto P\left(\Theta ,Y|w\right)P\left(w|w^{g},\Sigma^{g}\right)P\left(w^{g}\right)P\left(\Sigma^{g}\right), \end{array}$$

and likelihood,

(6) $$P(\Theta ,Y)|w)=\prod_{i=1}^{N}\prod_{t=1}^{T}p_{ti}^{y_{i}^{t}}{\left(1-p_{ti}\right)}^{\left(1-y_{i}^{t}\right)},$$

where *p*_*ti*_ is the probability respondent *i* chooses to swerve in scenario *T*, given by Equation 2. Kim et al. define a prior over the covariance matrix Σ^*g*^ as a LKJ covariance matrix with parameter *η* = 2,

(7) $$\begin{array}{l} \Sigma^{g}\sim LKJ\left(\eta \right), \end{array}$$

and specify a prior over the group weights,

(8) $$\begin{array}{l} w^{g}\sim N_{D}\left(0,\Sigma^{g}\right). \end{array}$$

Kim et al. continue to infer the model's individual weights, group norms and covariance matrices under the MAP estimate. We use Markov Chain Monte Carlo (MCMC) techniques to infer the posterior distributions over the model parameters. Making assumptions is essential when building data models, Bayesian methodology makes this explicit using priors. However, without conjugate priors inference becomes intractable in these models and we have to rely on approximative inference. In this paper we use MCMC methods which are correct in the limit. This paper looks closely at prior assumptions questioning if priors are chosen for computational simplicity or because they make sense.

## 3. Results

### 3.1. Preliminary Results

**Figure 1:**
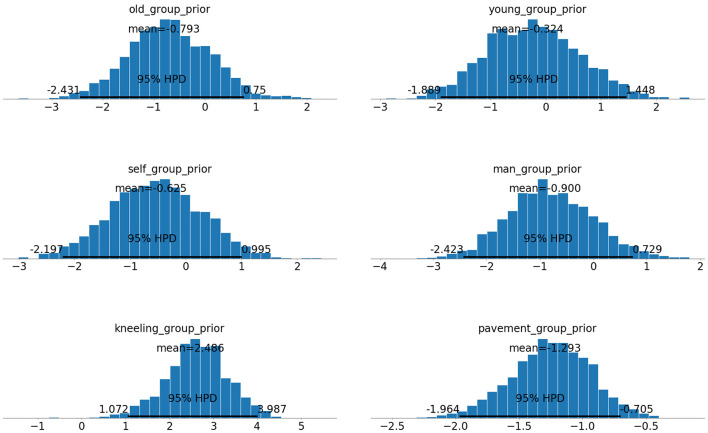
Inferred posterior distributions for group norm model parameters. **Top Left:** elderly, **Top Right:** infancy, **Middle Left:** self-preservation, **Middle Right:** middle-aged, **Bottom Left:** kneeling, **Bottom Right:** pedestrian.

One advantage of using MCMC is its ability to approximate posterior distributions for model parameters. The posterior distributions for the model's group norm parameters over 5,000 samples, with 1,000 tuning samples is shown in Figure 1.

As these posterior distributions closely resemble normal distributions, MCMC techniques can be used to derive parameter point estimates. Kim et al. use point estimates to discuss the success of their model in inferring moral principles. Kim et al. compare the accuracy of their model in predicting individual responses against a benchmark model, Benchmark 1, which models respondent values along the abstract moral principles Λ. In Benchmark 1, Kim et al. model the group weights,

(9) $$\begin{array}{l} w^{f}\sim Normal_{D}\left(\mu ,\sigma^{2}I\right), \end{array}$$

ignoring hierarchical structure and assume that inferring the moral preferences of an individual does nothing to inform the inference of others. This work introduces Benchmark 2 that models the individual weights,

(10) $$\begin{array}{l} w_{i}^{l}\sim \left[1,1,1,1,1,1\right], \end{array}$$

such that an individual assigns equal weight to each abstract principle.

**Figure 2:**
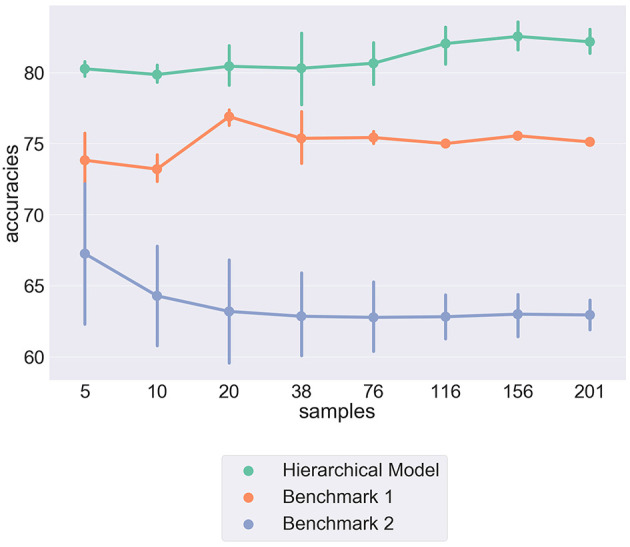
Pointplot showing the differences in predictive accuracies between three different models. The Hierarchical model is the implemented model detailed above, Benchmark 1 shows results from the flat model, Benchmark 2 shows results from a model that assumes all individuals have equal weights overall moral principles. The Y-axis shows predictive accuracy over individual decisions. The X-axis represents the number of respondents in sample. The vertical bars show variance from cross-validation.

Figure 2 shows the comparative predictive accuracies of our implemented model when compared to the two proposed benchmarks. We can see that the hierarchical model outperforms both benchmarks in predicting out of sample individual responses. The comparative success of both Benchmark 1 and the Hierarchical model, when compared to Benchmark 2, seems to provide experimental evidence to support the existence of moral principles that are unique to individuals and govern the judgements we make in moral dilemmas.

### 3.2. Importance of Assumptions

#### 3.2.1. LKJ Covariance Prior

**Figure 3:**
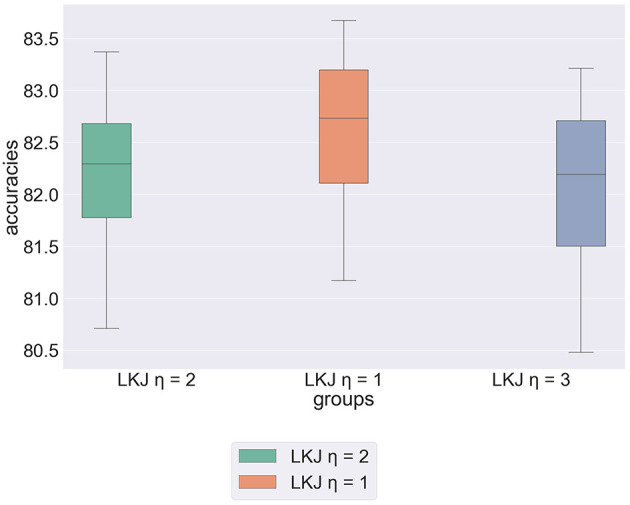
Box plot showing results of experimentation with the parametrisation of the LKJ covariance prior.

The model of Kim et al. uses the LKJ Cholesky Covariance prior distribution,

(11) $$\begin{array}{l} \sum^{g}\;\sim \;LKJ\left(\eta =2\right), \end{array}$$

to define abstract moral weights, where η controls the level of matrix correlation (Lewandowski et al., 2009). However, there is little evidence that accounts for the extent as to which moral principles are related to each other (Clouser and Gert, 1990). We propose experimentation with the LKJ distribution varying η to ascertain the distribution that best suits the structure of the underlying data. Figure 3 shows the results of our experimentation varying η. The boxplot shows that the model achieves greatest predictive accuracy when η = 1. In other words, the model achieves higher accuracy when constraints on correlation are weakened. This implies our earlier hypothesis, that Kim et al.'s prior distributions were chosen primarily for practicality rather than as a result of extensive evaluation.

#### 3.2.2. The Half Cauchy Distribution

Kim et al. model the standard deviations for the multivariate Gaussian distribution, as normally distributed with low variance. We propose a weaker prior distribution to challenge the idea that individual moral principles are tightly clustered around a group norm and that group norms are constrained to a narrow range. Gelman identified that many distributions historically used for non-informative priors introduce levels of subjectivity into results (Gelman, 2006). Furthermore, Gelman proposes the half Cauchy density as a reference non-informative prior that should be used for the standard deviation term in a hierarchical model. The half Cauchy is a special case of the conditionally-conjugate folded-non central-t family of prior distributions for variance (Gelman and Hill, 2006). It has a wide peak at 0 and a scale parameter *A*. As *A* tends toward infinity, the half Cauchy distribution becomes a uniform prior on the standard deviation. Large and finite values of *A* represent a weak prior as the distribution has a gentle slope in the tail. We propose modeling the standard deviations of the group-norm parameters and individual-weights using the half Cauchy distribution,

(12) $$\begin{array}{l} \sigma \sim halfCauchy\left(10\right). \end{array}$$

**Figure 4:**
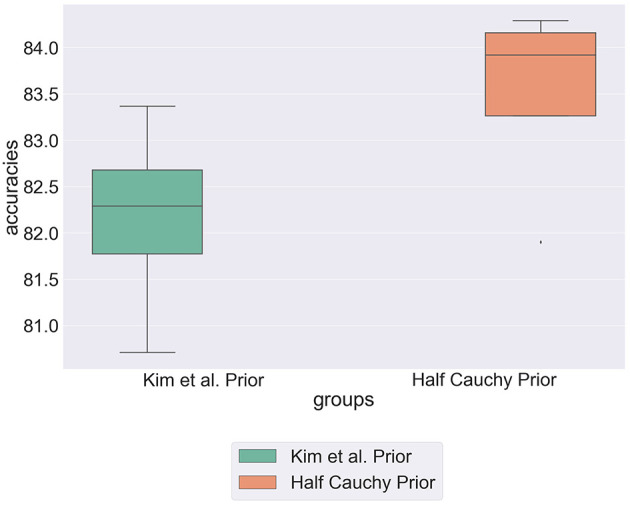
Box plot showing predictive accuracy of the hierarchical model, comparing modeling the standard deviation hyperprior as a narrow normal distribution and a half Cauchy distribution.

Figure 4 shows the comparative predictive accuracies between the standard deviation hyperprior specified by Kim et al. and the half Cauchy hyperprior. We can see that the half Cauchy prior statistically significantly improves the predictive accuracy of the model. This re-enforces our hypothesis that Kim et al.'s prior assumptions are too specific to represent the data. Further, there is no conclusive evidence to suggest that individual moral beliefs are concentrated around a central group value. We therefore propose modeling individual weights with a generalized distribution that has increased mass in its tails.

#### 3.2.3. The T Distribution

In their work “Student-t Processes as Alternatives to Gaussian Processes” (Shah et al., 2014), Shah et al. highlight the popularity of the normal distribution due to its interpretability, large support base and the success of its empirical results. They continue to define Student-t processes as a family of elliptical processes that generalize the normal distribution,

(13) $$\begin{array}{l} {\left(f\left(x_{1}\right),\ldots ,f\left(x_{n}\right)\right)}^{T}\sim MVT_{n}\left(v,\phi ,K\right), \end{array}$$

where *K* ∈ Π(*n*) is the covariance matrix and ϕ ∈ ℝ^*n*^ is the mean vector (Shah et al., 2014). Shah et al. stress the importance of the *v* parameter which controls the heaviness of distribution tail. As *v* increases, the Student-t distribution converges to a normal distribution. By using a distribution with more mass in the tails we are weakening the assumption on the model parameters, relaxing the constraint to be concentrated around a central value. We therefore propose to model the group norms and individual weights using the Student-t distribution,

(14) $$\begin{array}{l} w^{g}\sim MVT\left(5,0,\Sigma^{g}\right), \end{array}$$

(15) $$\begin{array}{l} w^{i}\sim MVT\left(5,w^{g},\Sigma^{g}\right). \end{array}$$

**Figure 5:**
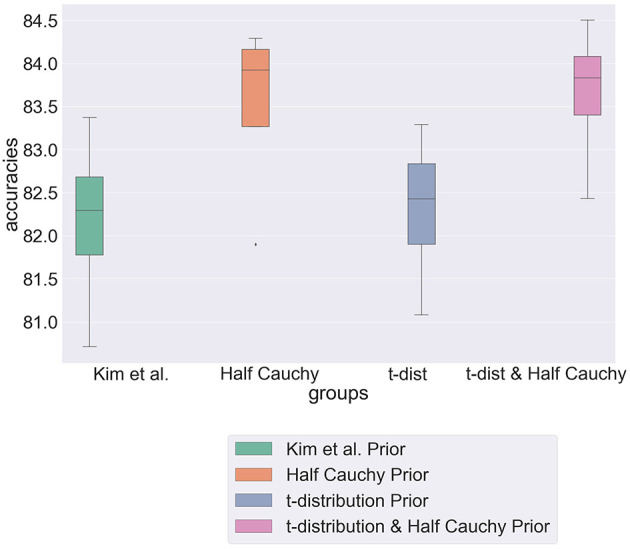
Box plot showing predictive accuracy of hierarchical model when modeled with different prior distributions.

Figure 5 is a box plot showing the predictive accuracy of the hierarchical model, constructed from the method of Kim et al., under the novel parametrisations presented in this section. It shows that whilst the combined model achieves greatest predictive accuracy over a single partition, the half Cauchy model remains the most robust with the lowest variance value across partitions. These results demonstrate the difficulty in selecting a prior distribution for a model. Is it more important to have a consistently performing model or achieve the greatest predictive accuracy? However, through demonstrating the sensitivity of the model to parameterisations, we have motivated the importance of carefully considering prior distributions, particularly in cases where prior knowledge is scarce. Furthermore, in the context of morality we must be careful to view the results in light of our dataset and assumptions, stressing the importance of further evaluation to generalize results.

### 3.3. Utilitarianism vs. Deontology

Kim et al. use utility calculus to compute welfare-trade offs that determine individual moral decisions. Quantifying weights over moral principles that are then summed to determine an overall utility is synonymous with the ethical perspective of utilitarianism and places a heavy assumption over the ways in which humans make moral decisions. We continue to raise questions over the use of utility functions to infer individual moral principles and attempt to ground the experimental results found by our model in ethical theory.

Historically, researchers have identified utilitarianism and deontology as the two central competing modes of moral decision making (Gray and Schein, 2012). Ditto and Liu discuss the differences between both perspectives and attempt to explain how they both contribute to moral decision making (Ditto and Liu, 2016). Ditto and Liu conjecture that humans tend to balance utilitarian outcomes with deontological principles (Ditto and Liu, 2016). For example, if an individual believes it is wrong to kill children and they are presented with a scenario that forces them to choose between saving five men or one child, this individual would increase their weighting of the child to be in line with their deontological standing. We incorporate the perspective of Ditto and Liu into the model proposed by Kim et al.

As part of the German Autonomous Vehicle study (Faulhaber et al., 2018), participants were asked a series of questions following their completion of the moral dilemma trials. These questions are intended to gauge the respondent's perspective on general ethical questions, i.e., “Protecting oneself should have priority over protecting others.” Participants are asked to give each statement a score ranging from one to seven where seven indicates strong agreement with the statement. These questions obtain a sense of the individual's view of the rightness or wrongness of the action, when removed from a specific context, which can be equated to their deontological perspective. We therefore propose to take the quantification of a participant's deontological beliefs and combine this into the model such that an individual's weights are modeled,

(16) $$\begin{array}{l} w_{i}\sim N\left(\left(w^{g}+\phi \right)/2,\Sigma^{g}\right), \end{array}$$

using ϕ to represent the vector of individual *i*'s deontological beliefs.

**Figure 6:**
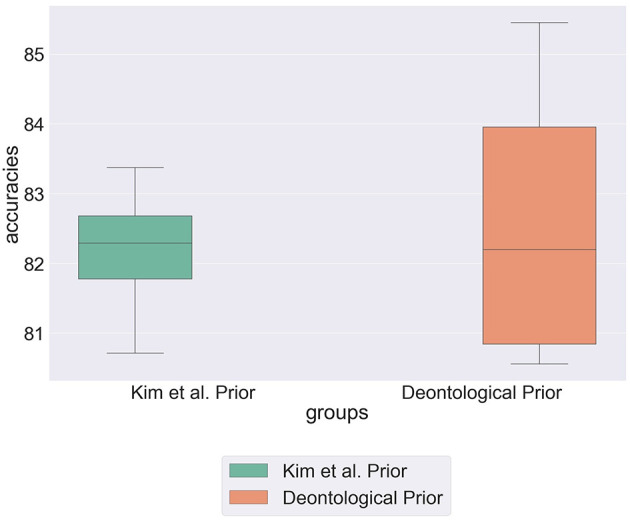
Box plot showing predictive accuracy of the hierarchical model with priors specified by Kim et al. (Kim et al., 2019) when compared to the model initialised with deontological priors.

Figure 6 shows that by incorporating deontological statements into prior distributions the model achieves its highest recorded predictive accuracy of 85.5%. This supports Ditto and Liu's hypothesis that deontological statements are intrinsically linked to how we weight alternatives in moral dilemmas (Ditto and Liu, 2016). However, the high variability across the partitions for this parametrization suggests that this is not true for all individuals and the high variability within results refers to the extent in which individuals use deontological rules to shape their actions.

### 3.4. Cross Domain Model

We have seen how the model proposed by Kim et al. has provably inferred moral principles over two alternate datasets but we have only considered moral dilemmas in the context of the Trolley Problem. Awad claims that trolley dilemmas are too simple, too rare, and unrealistic to be used as framing mechanisms (Awad, 2017). We do not, therefore, pretend to have implemented a model capable of extracting the full spectrum of human morality. However, we have used the Trolley Problem as a framing device to show the feasibility of this goal. We now present the application of Kim el.'s model to more general moral dilemmas.

#### 3.4.1. Transferring Model to a New Domain

A study conducted by Haykawa et al. investigates the contribution of foreign language in responses to moral dilemmas (Hayakawa et al., 2017). Hayakawa et al. present 20 variations of complex moral scenarios to participants, gathering their opinions on moral dilemmas ranging from coma patients to bear attacks. These moral dilemmas from Hayakawa et al. are more complex and realistic than those in the Faulhaber et al. study. Hayakawa et al. present six experiments, each containing a slight variation in dilemmas, to a sample population size of 224 in a laboratory environment. We now present the application Kim et al.'s methodology to the data generated by the complex moral scenarios of Hayakawa et al. One of the biggest challenges in transferring the model of Kim et al. to the new domain is redesigning the mapping of character vectors to the abstract feature space. Indeed, due to the high variability between the 20 moral dilemmas presented in the experiments of Hayakawa et al. the resulting abstract feature vectors are much more difficult to design. We therefore experiment with different abstract feature vectors, encapsulating different numbers of moral feature parameters.

**Figure 7:**
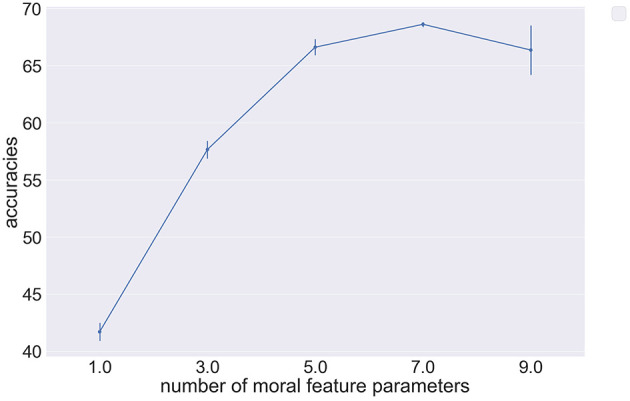
Pointplot showing the relationship between model predictive accuracy and the number of abstract moral features used in parametrisation on the Hayakawa et al. dataset (Hayakawa et al., 2017).

After defining the abstract feature vectors, we use the model parametrization of Kim et al. We are then able to infer individual moral preferences over respondents and use these to predict out of sample decisions. Figure 7 shows the predictive accuracy of our implemented model using abstract feature vectors of varying length to represent Hayakawa et al.'s dataset. Figure 7 shows how the predictive accuracy of the model increases with the number of abstract moral features used to describe underlying moral dilemmas, where error bars represent the variance in accuracy over different permutations of chosen abstract features. Our results show that the accuracy decreases as the number of parameters exceeds seven. Furthermore, when we attempt to define eleven abstract moral features the model encounters sampling issues and does not converge. This behavior can be explained by the incapability of the model in dealing with sparse abstract feature vectors. Furthermore, we cannot conclude that seven abstract moral features are sufficient to describe individual moral preferences. In fact, these results show the impact of placing different assumptions over how many abstract features are used to describe moral dilemmas and represents a significant challenge to the method of Kim et al. if the model is to be expanded to learn complex moral dilemmas.

## 4. Discussion

This work has replicated the exciting results produced by state of the art in modeling ethical decision making. We have extended these studies by combining philosophical and mathematical theory to suggest alternative model parametrisations, that both reflect ethical theory more closely and improve quantiative results. However, above all we have raised a multitude of potential barriers of this approach to automating ethical decision making. Further, this work has established the enormity of the ethical AI challenge and has throughout emphasized the importance of questioning quantitative results. One key challenge this work has uncovered is the challenges of interpreting machine learning results based on data that describes tacit human knowledge. Results can only be interpreted relative to their associated assumptions and making assumptions of morality is very challenging. The richness of this work therefore lies in its cautionary rhetoric and how we apply the presented analytical techniques to future work. We therefore conclude by presenting some possible directions for future work.

### 4.1. Oversimplification of Decision Making

One significant criticism of the implemented model is the decision to model decision making as a linear combination of moral preferences, remaining invariant over time. We question whether a richer model of moral decision making would be achieved by modeling, not just the decision outcome, but the cognitive process of deliberation. Busemeyer et al. identify the shortcomings of decision making models that use expected utility theory (Busemeyer and Townsend, 1993). They draw on the psychology theory of James (James, 2013), to highlight the importance of cognitive deliberation processes in making decisions under uncertainty. Busemeyer et al. build on historic static models of decision making to propose a dynamic model, using a drift diffusion model to show how preference relations change as a function of deliberation time.

An alternative oversimplification of decision making assumed by the Kim et al. model is the decision to model decision making processes as utility functions representing underlying moral preferences. Furthermore, there has been huge debate within ethics over “Principilism,” the practice of using principles to replace moral theories. Clouser and Gert argue that misguided moral principles obscure moral reasoning by misrepresenting and over simplifying moral theories (Clouser and Gert, 1990). We can see this oversimplification of moral theories within our implementation. When defining their utility calculus, Kleiman-Weiner et al. include both a linear representation of moral preferences and another term representing other abstract individual qualities that affect moral decision making such as empathy. Furthermore, when defining their model, Kim et al. ignore this additional term and focus on inferring weights over moral principles. An extension of this model would therefore be incorporating a method of modeling the additional source of moral valuation.

Considering empathy as a factor that shapes moral decision making in the Trolley Problem has been approached in the literature by Wilson and Scheutz who begin by modeling decision making as utility scores, a linear combination of propositions that have an associated weight (Wilson and Scheutz, 2015). To account for empathy in their model, Wilson et al. introduce a new weight on the utility of a proposition that determines the empathetic response of an action. This empathy model could be used to enrich the model of Kim et al. by accounting for individuals whose behavior could not be explained by moral principles alone.

### 4.2. Toward a Hybrid Model

This work discusses the difference between bottom-up and top-down approaches to engineering moral machines. We have presented a bottom-up approach to learning human moral judgments. The next stage in engineering an ethical machine would therefore be to incorporate ethical or legislative rules into the system presented in this work to produce a hybrid ethical model. Noothigattu et al. recommend that ethical rules could control behavior of the machine. However, they claim it impossible to specify ethical rulings that cover every possible scenario (Noothigattu et al., 2017). Loreggia et al. propose an alternative method for encoding ethical rules within models of human moral behavior (Loreggia et al., 2018). Their proposed system uses CP-nets to evaluate whether human preferences are compatible with specified ethical principles. CP-nets were presented by (Boutilier et al., 2004) as a graphical representation of preference data and allow for the qualitative instead of quantitative assessment of preferences using preference relations (Boutilier et al., 2004). Loreggia et al. continue to construct two alternative CP-nets, with the first over human moral preferences and the second over ethical principles. This formulation allows Loreggia et al. to calculate the distance between both CP-nets and a way of measuring whether moral principles are “close enough” to ethical rules (Loreggia et al., 2018). However, questions remain over what set of ethical rules should be used. We propose the use of the recently defined German ethical guidelines for autonomous vehicles commissioned by the German federal government and the first of its kind to standardise the behavior of autonomous vehicles in ethical dilemmas (Transport and Infrastructure, 2017). The incorporation of these guidelines as ethical rules would show the difference between human behavior and legal requirements defined by lawyers which could make for a very interesting comparison.

### 4.3. Quality of Data

An argument that uses inductive reasoning can never be proven right or wrong but can be strengthened through use of quality evidence. This means that we need lots of representative data to justify our method. We continue to re-iterate that the results and arguments presented in this work are contextualized to our datasets and are by no means a resolution to the challenge of designing moral machines. Furthermore, future work must continue to gather quality data. One specific issue surrounding the quality of data used by this study is lack of fully representative data. Whilst the Moral Machine dataset (Awad et al., 2018), collects responses from all over the world, the German Autonomous Vehicle dataset (Faulhaber et al., 2018) only represents the moral beliefs of Western cultures. If future studies wish to use models of morality to automate ethical decision-making, consideration must be made to ethnic and socioeconomic groups that are traditionally under represented. Importantly, through this work we have highlighted the challenges of generating enough quality data surrounding morality which must be addressed if future inductive models are to be trusted.

### 4.4. Algorithmic Fairness

Currently, one controversial area within ethical artificial intelligence is the concept of algorithmic fairness. A 2016 study found that the algorithm used in criminal sentencing, to assess risk of recidivism, was displaying signs of racial bias (Angwin et al., 2016). The fact that artificial intelligence has already spread to areas in which it has a big impact on human lives means that developers must start thinking beyond the predictive accuracy of their systems and consider the social implications (Kusner et al., 2017). Learning a model of human morality is inherently biased. We have seen how our data population are likely to place higher preference on humans with certain characteristics. Is it therefore ethical to use models of human morality in automating ethical decision making? Many critics would argue no, that humans are not an example of ethicality and that ethical intelligent systems should be designed without individual moral influence. Advocates, however, would argue that ethics is a human construct and therefore can only be considered within human conduct. If we are to use human moral behavior to automate decision making, extensions to models must be made to prevent the use of protected attributes in decision making (Kusner et al., 2017). In their paper, “Counterfactual Fairness,” Kusner et al. describe protected attributes as variables that must not be discriminated against, relative to a particular system. Kusner et al. present counterfactual fairness as the idea that a decision is only fair if it is fair in both the real world and a counterfactual world, where the target individual belongs to an alternative demographic group.

The results of the implemented model show that, within our data population, individuals place a higher weight on the infancy moral principle when compared to the elderly moral principle. This result shows that individuals are likely to discriminate based on potentially protected attributes, such as age. The ability of the model to infer quantified rankings over moral principles could, potentially, be expanded to recognize certain biases in certain populations, or software. An interesting extension of this project would be to apply the model of Kim et al. to the COMPAS open source dataset used for the recidivism study (Angwin et al., 2016). By assigning a weighting over race moral principles, we hypothesize that the model would be able to extract racial bias. A model capable of verifying the neutrality of a piece of software or a person would be invaluable in industries such as law, banking and recruitment.

## Author Contributions

TS is responsible in entirety to the design and implementation of the research, to the analysis of the results and to the writing of the manuscript.

### Conflict of Interest Statement

The author declares that the research was conducted in the absence of any commercial or financial relationships that could be construed as a potential conflict of interest.
